# Glucosuria in Paludal Fever

**Published:** 1860-04

**Authors:** 


### *Glucosuria in Paludal Fever.*

Burdel, of Vierzon, has addressed a paper on this subject to the French Academy of Sciences. The results of his researches are thus stated:

1. In paludal fevers there exists a true diabetes or glucosuria.

2. This glucosuria is only ephemeral; that is to say, being the indication of derangements in the organism, it appears with the fever, persists during its continuance, and disappears with it.

3. Glucosuria in paludal fever shows clearly the existence of a special agency destroying the equilibrium existing between the cerebro-spinal and sympathetic systems.

4. This explanation of Claude Bernard is confirmed by the following facts: The more violent the access, the more intense the chill, the larger, also, is the quantity of sugar in the urine—on the contrary, when the attacks have been frequent and have lost their force, and, in a word, the more a cachexy is established, the less sugar is produced.—*Gazette des Hôpitaux.*

